# Abnormal late visual responses and alpha oscillations in neurofibromatosis type 1: a link to visual and attention deficits

**DOI:** 10.1186/1866-1955-6-4

**Published:** 2014-02-21

**Authors:** Maria J Ribeiro, Otília C d’Almeida, Fabiana Ramos, Jorge Saraiva, Eduardo D Silva, Miguel Castelo-Branco

**Affiliations:** 1 Visual Neuroscience Laboratory, Institute for Biomedical Imaging and Life Sciences (IBILI), Faculty of Medicine, University of Coimbra, Azinhaga de Santa Comba, Coimbra 3000-548, Portugal; 2 Medical Genetics Department, Pediatric Hospital of Coimbra, Coimbra, Portugal

**Keywords:** Visual evoked potentials, Alpha rhythm, Contrast response function, Electroencephalogram (EEG), Neurofibromatosis type 1 (NF1), Paediatric

## Abstract

**Background:**

Neurofibromatosis type 1 (NF1) affects several areas of cognitive function including visual processing and attention. We investigated the neural mechanisms underlying the visual deficits of children and adolescents with NF1 by studying visual evoked potentials (VEPs) and brain oscillations during visual stimulation and rest periods.

**Methods:**

Electroencephalogram/event-related potential (EEG/ERP) responses were measured during visual processing (NF1 n = 17; controls n = 19) and idle periods with eyes closed and eyes open (NF1 n = 12; controls n = 14). Visual stimulation was chosen to bias activation of the three detection mechanisms: achromatic, red-green and blue-yellow.

**Results:**

We found significant differences between the groups for late chromatic VEPs and a specific enhancement in the amplitude of the parieto-occipital alpha amplitude both during visual stimulation and idle periods. Alpha modulation and the negative influence of alpha oscillations in visual performance were found in both groups.

**Conclusions:**

Our findings suggest abnormal later stages of visual processing and enhanced amplitude of alpha oscillations supporting the existence of deficits in basic sensory processing in NF1. Given the link between alpha oscillations, visual perception and attention, these results indicate a neural mechanism that might underlie the visual sensitivity deficits and increased lapses of attention observed in individuals with NF1.

## Background

Neurofibromatosis type 1 (NF1) is the most common single gene disorder that affects brain function [1]. Impairments include deficits in visual perception, motor and visuomotor skills, language, memory, attention and executive function [1,2]. The neural mechanisms underlying brain dysfunction are likely to involve both neurochemical and structural alterations [2-5].

Deficient visually-evoked activation of occipital, temporal and parietal brain regions have been shown by functional magnetic resonance imaging (fMRI) studies [6,7]. Occipital brain regions encompass early visual cortical areas that underlie low-level vision. Impairments in these areas may therefore result in poor processing of low-level stimulus features, for example contrast, color, size, texture or motion. In fact, recently we have shown that the response to visual contrast is abnormal in individuals with NF1 [8]. This deficit was reflected in reduced chromatic and achromatic contrast sensitivity. Interestingly, chromatic contrast sensitivity was significantly affected when testing sensitivity for red-green contrast but was relatively spared for blue-yellow contrast, suggesting specific deficits within the parallel detection mechanisms that subserve low-level vision (red-green, blue-yellow and achromatic) [9]. The achromatic channel is highly sensitive to low-spatial and high-temporal frequencies and plays an important role in spatial localization and motion processing [10]. The red-green channel underlies fine discrimination of visual features, particularly in the central visual field [11]. The function of the blue-yellow mechanism is less understood but it certainly plays a role in color vision, spatial processing and motion perception [12-14]. Thus, specific deficits in each of these independent pathways might have specific implications in the visual phenotype of these patients.

One possibility is that the contrast response functions of the visual cortex of these patients are abnormal leading to poor sensitivity to stimuli with low contrast. To test this hypothesis, we recorded the electroencephalogram (EEG) of children and adolescents with NF1 during stimulation with visual stimuli of various contrasts. Different contrast levels were used because deficits in visual processing in NF1 may be dependent on contrast level, as suggested by our previous behavioral study [8]. Indeed, for certain neurologic populations, deficits in visual cortical activation depend on stimulus contrast [15]. In addition, we used specific visual stimuli designed to bias the activation of the achromatic, red-green or blue-yellow mechanisms [9,16,17].

EEG recordings enable the study of cortical evoked potentials, including visual evoked potentials (VEPs), and also cortical oscillatory activity. Two types of brain oscillations, detected over the parieto-occipital cortex, have been associated with the neural processing of visual stimuli: the alpha (8 to 13 Hz) and gamma oscillations (30 to 90 Hz). These relate with visual processing in an opposite way, with high amplitude of alpha waves associated with decreased excitability of the visual cortex and high amplitude of gamma oscillations associated with neural processing and encoding of visual stimuli [18]. Importantly, abnormal oscillatory activity, related with impaired visual processing, has been observed in disorders affecting the nervous system, such as attention deficit hyperactivity disorder (ADHD), autism and schizophrenia [19-21], and thus might also be linked with the deficits observed in NF1. Furthermore, in normotypical adults, higher pre-stimulus amplitude of alpha oscillations has been associated with poorer visual detection [18]. With this in mind, we sought to determine if NF1 performance in a visual detection task could be related with abnormal alpha oscillations. In addition, as excitability of the visual cortex is related with the neural oscillatory state, in particular to the alpha rhythm [18], we characterized, in a subgroup of patients and controls, the amplitude of brain oscillations at rest (during periods with eyes closed and eyes open) to determine if baseline cortical excitability was affected in NF1.

## Methods

### Participants: recruitment, exclusion criteria and group characteristics

Children and adolescents with NF1 were recruited in collaboration with the Genetics Department of the Pediatric Hospital of Coimbra in Portugal. All participants met the National Institutes of Health Consensus Development Conference clinical criteria for NF1 [22]. We excluded patients with known brain pathology or ophthalmological problems that could influence the results (for example amblyopia). Furthermore, in order to ensure that the patients included in the study had no unknown brain pathology (for example optic gliomas), they were submitted to magnetic resonance structural scans (magnetization-prepared rapid acquisition of gradient echo (MPRAGE) and fluid-attenuated inversion recovery (FLAIR) sequences). Standard neuroradiological assessments were carried out by an experienced neuroradiologist. Only children and adolescents with NF1 but no significant structural anomalies, besides T2-hyperintensities, were included in the study.

In addition, all patients were submitted to a complete ophthalmic examination, including best-corrected visual acuity, stereopsis evaluation, slit lamp examination of anterior chamber structures and fundus examination. Lisch nodules were observed in a subset of individuals but no anomalies that could affect vision were found.

For the control group, we recruited typically developing participants from a local school. These participants had no history of learning, developmental, cognitive, neurological or neuropsychiatric problems.

For analysis, we included 17 patients and 19 control children and adolescents. The EEG file of one participant with NF1 recorded during the achromatic stimulation experiment was corrupted leaving 16 participants with NF1 in the analysis of the response elicited by achromatic stimulation. For the second part of the protocol (analysis of alpha amplitude under eyes open and eyes closed conditions), only a subgroup of participants were available to participate (NF1 n = 12; control n = 14). The age and sex ratios of the two groups were not significantly different (*t*-tests were used for age comparisons and chi-square tests for sex ratio comparisons), both for the groups tested in the visual stimulation experiment (mean age ± standard deviation (age range) in years: NF1 = 11.9 ± 2.3 (8 to 17), control = 12.9 ± 2.6 (8 to 17); sex ratio (F/M): NF1 = 12/5, control = 11/8) and for the subgroups tested in the eyes open/eyes closed experiment (mean age ± standard deviation (age range) in years: NF1 = 12.7 ± 2.0 (10 to 16), control = 13.1 ± 2.3 (10 to 17); sex ratio (F/M): NF1 = 9/3, control = 9/5).

The genetic and neuropsychological characterization of this group of children and adolescents with NF1 was reported in our previous study [8]. We administered the Portuguese adapted version of the Wechsler Intelligence Scale for Children (WISC-III), in all participants with NF1 and in a subgroup of control children and adolescents (n = 8). The mean (standard deviation) full-scale IQ for the NF1 group was 97 (16), while for the subgroup of control participants it was 124 (17).

Four of the patients with NF1 had been previously diagnosed with ADHD and were managed with stimulant medication (methylphenidate). These children were not given the medication on the days of testing, ensuring that they were not under the influence of methylphenidate during testing.

### Protocol approvals and patient consents

The study was conducted in accordance with the tenets of the Declaration of Helsinki and was approved by the Ethics Committees of the Faculty of Medicine of Coimbra and of the Children’s Hospital of Coimbra. Written informed consent was obtained from the legal representatives of the participants, after explanation of the nature and possible consequences of the study. In addition, all participants gave written or oral informed consent.

### Visual stimulation

The visual stimuli used were adapted from our previous study [23]. Stimuli were generated with MATLAB (R2008a, MathWorks, Natick, MA, USA) and presented with the stimulation software STIM^2^ (Neuroscan, Charlotte, NC, USA) with a display resolution of 1,280 × 1,024 × 32 and graphic processing unit NVIDIA GeForce 6600, provided by Neuroscan. The stimuli were presented in a CRT monitor (Diamond Digital color monitor, Mitsubishi Electric Australia, Rydalmere, NSW, Australia) with the refresh rate set at 85 Hz.

**Figure 1:**
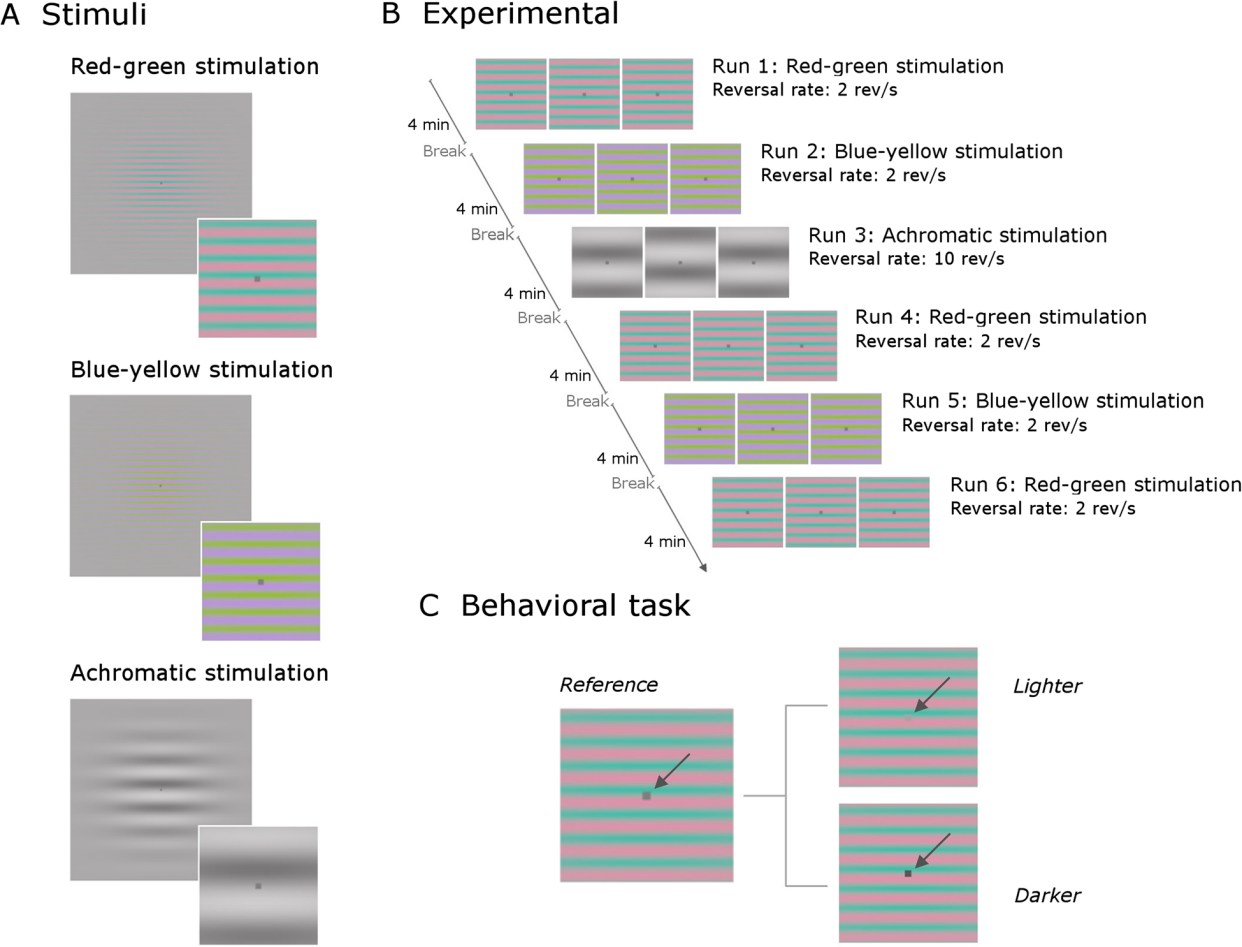
**Schematic diagram of the visual stimuli, experimental procedure and behavioral task. (A)** Schematic diagram of visual stimuli used, with magnified insets centered on the fixation square. **(B)** Experimental protocol used for measurements of visual evoked potentials (VEPs). **(C)** The three different levels of luminance of the fixation square, shown here on the red-green stimulus. The participants had to detect a brief change in the luminance of this square from the reference luminance to a lighter or darker grey.

Stimuli were circular horizontal Gabors (sinewave gratings modulated by a Gaussian window) presented in phase reversal mode at the centre of the CRT monitor (Figure 1A). Stimuli diameter, defined as two times the standard deviation of the Gaussian aperture filter, was 12° of visual angle. The viewing distance was 1 m and the screen subtended a visual angle of 21° in width and 16° in height. Stimuli chromatic calibration was achieved by the procedure described before [23]. The achromatic stimuli were composed of luminance modulations. The chromatic stimuli were isoluminant (red-green or blue-yellow) composed of only chromaticity modulations. The mean luminance of the stimuli and background was 39 cd/m^2^ with the Commission Internationale de l’Éclairage (CIE 1931) x- and y-coordinates x = y = 0.29. Stimulus chromaticities were defined, as in our previous study [23], using a three-dimensional cone contrast space in which each axis represents the activation of the long-wavelength (L), middle-wavelength (M) and short-wavelength (S) cone types, normalized with respect to the white background (cone contrast) [24-26]. These three cone types are the photoreceptors present in the human retina responsible for color vision. Stimulus contrasts were calculated as the length of the vectors in cone contrast space and expressed as a percentage of the maximum contrast used for each stimulus type. The CIE x- and y-coordinates of our stimuli were as follows: background and achromatic stimuli, x = y = 0.29; +S cone, x = 0.26, y = 0.21 and –S cone, x = 0.36, y = 0.51; L-M cone, x = 0.33, y = 0.28 and M-L cone, x = 0.23, y = 0.32.

To enhance the relative isolation of the different detection mechanisms, we used stimuli with distinct spatiotemporal criteria. Differences in temporal rates are necessary due to the need to differentially recruit neural populations with different tuning properties: the achromatic channel is highly sensitive to low-spatial and high-temporal frequencies, while the two chromatic channels, red-green and blue-yellow, are more sensitive to low-temporal and high-spatial frequencies.

Achromatic stimulus parameters: spatial frequency, 0.5 cycles per degree (cpd); reversal rate, 10 reversals per second (rev/s; full cycling rate, 5 Hz).

Red-green and blue-yellow stimuli parameters: spatial frequency, 2 cpd; reversal rate, 2 rev/s (full cycling rate, 1 Hz).

### Experimental protocol: visual evoked potential measurements

During EEG recording, stimuli presentation was divided into runs of around 4 minutes. Participants were allowed to rest in between runs as necessary. During each run, only one type of stimulus was presented (achromatic, red-green or blue-yellow stimulation). Within the run, stimulus contrast changed randomly every 3 seconds. We recorded two 4-minute segments probing the blue-yellow mechanism, three 4-minute segments probing the red-green mechanism and one 4-minute segment probing the achromatic mechanism. We recorded more trials in the red-green condition because the maximum chromatic (cone) contrast used for the red-green stimuli was considerably smaller than the chromatic contrast used in the blue-yellow stimulation, resulting in reduced signal-to-noise in this condition. This difference in chromatic contrast was unavoidable due to the constraints resulting from the isoluminance requirements. Increasing the number of trials increased signal-to-noise ratio in this condition. The achromatic stimulation with its higher temporal frequency (5× more) allowed the inclusion of more trials in a smaller time window. The order of the runs was fixed for all participants: red-green, blue-yellow, achromatic, red-green, blue-yellow and red-green (Figure 1B). In total, for the achromatic, red-green and blue-yellow conditions, we recorded 420, 306 and 210 phase-reversals per stimulus contrast, respectively. The choice of the number of trials recorded per condition was based on our previous study on within condition comparisons with these type of stimuli [23].

The participants sat comfortably 1 m from the computer screen and were requested to fixate a small grey square on the centre of the screen (width = 0.16° of visual angle). In order to help maintain fixation and to keep the participant’s attention stable throughout the EEG recording session, the children were engaged in a detection task involving the detection of a luminance change of the central fixation square that occurred at intervals with randomly defined durations with a minimum of 3 seconds and a maximum of 10 seconds. This procedure enabled the assessment of fixation reliability by generating a sufficient number of trials where correct responses were only possible when fixation was on the central square. During EEG recording, the luminance of the square changed either to a higher value or to a lower value and after 500 ms the square’s luminance returned to its initial value (Figure 1C). The participants were requested to detect this brief change and report through button presses the type of polarity change in luminance: with left index finger if the change was to a lighter square (luminance increase) or with right index finger if change was to a darker square (luminance decrease). An initial training period ensured all participants understood the task and were able to discriminate between the different levels of luminance. The luminance differences used were above detection threshold for all participants.

Although the detection task was superimposed on the visual stimuli of interest, we believe these events did not significantly affect the measured VEPs because the number of times that the fixation square changed luminance during the EEG recording was small in comparison with the number of contrast phase reversals (less than 8% of the times for red-green and blue-yellow stimulation and less than 2% for the achromatic stimulation).

### Experimental protocol: eyes-closed and eyes-open resting conditions

The participants sat comfortably 1 m from the computer screen and alternated between 2-minute periods of eyes closed or eyes open. A brief (50 ms duration including 15 ms rise- and fall-times) 1,000 Hz 80 dB tone signaled when to close or open the eyes. The protocol lasted 12 minutes starting with a 2-minute eyes-closed period. During the eyes-open condition, participants were instructed to visually fixate the grey square presented on the centre of the screen in front of them showing the same grey background and fixation square used in the VEP measurements.

### Data acquisition and analysis

EEG signal was recorded from six parieto-occipital channels (PO3, POZ, PO4, O1, OZ and O2) using a 64-channel Neuroscan system with scalp electrodes placed according to the International 10–20 electrode placement standard and with reference between CPZ and CZ and ground between FPZ and FZ. The same reference channel was used for acquisition and data analysis. Acquisition rate was 1,000 Hz. Vertical and horizontal electrooculograms were recorded in order to correct and/or reject artifacts caused by blinking and eye movements. A trigger pulse was generated at the onset of each stimulus (at each phase reversal, during visual stimulation, or at the acoustic signal to close or open the eyes). Data analysis was performed with Scan 4.5 (Neuroscan).

We started the analysis by correcting the eye blinking artifacts present in the EEG recordings using an automated procedure available in Scan 4.5 that consisted of the following processing steps. First, an average of the EEG signal locked with eye blinks was created for every subject for each stimulation type (blinks were identified in the VEO channel as events where the EEG signal went below −100 μV). Using spatial principal component analysis on the average signal we extracted the spatial component topography and time series associated with the blinking artifact. Then, we filtered it out of the data, leaving the EEG signal with negligible eye blinking contamination.

On the EEG recordings corrected for eye blinking artifacts, we applied a bandpass filter with cutoff frequencies of 1 and 100 Hz and attenuation of 12 dB/octave. Filtering was performed using the Zero Phase Shift option available in Scan 4.5, consisting of the application of a forward Butterworth filter followed by a reverse Butterworth. Filtering twice, once in each direction, is important to null the effect of filtering on the evoked potential peak latencies.

In order to ensure that the artifact correction had indeed corrected the blink artifacts, all the continuous files were then visually inspected and periods with remaining eye blinking or other muscle artifacts were manually rejected.

The filtered files were cut into epochs. The epochs of the signal elicited by the achromatic stimuli were 600 ms long (six phase reversals), non-overlapping and starting at the beginning of a cycle (at the phase reversal). Given that we were measuring a steady-state response, the achromatic baseline was determined as the average value of the entire sweep ranging from stimulus onset until 600 ms after, that is, our baseline represents the mean amplitude of three temporal cycles of the achromatic stimulus. The epochs of the signals elicited by the red-green and blue-yellow stimuli were 500 ms long, starting 100 ms before phase reversal and finishing 400 ms after. Baseline was set from −100 ms to the onset of the stimulus (0 ms). Further artifact rejections were then conducted automatically on the basis of deflections with amplitude higher than 100 μV.

After artifact rejection, the average number of artifact-free epochs per contrast level remaining was: (mean ± standard deviation) achromatic stimulation, NF1 = 69 ± 1, CNT = 69 ± 1; red-green stimulation, NF1 = 294 ± 23, CNT = 301 ± 4 and blue-yellow stimulation, NF1 = 205 ± 4, CNT = 207 ± 3. There was no significant group differences concerning the number of epochs used (nonparametric comparison given the non-normal distribution of the data; Mann–Whitney U test, *P* >0.05).

For time-domain analyses, we averaged the VEP data across the six recorded electrodes (PO3, POZ, PO4, O1, OZ and O2) before peak analyses. For frequency analyses, spectral amplitudes and peak frequencies were calculated for each electrode and these data were then averaged across the six electrodes before statistical analyses. Pooling data across electrodes has the advantage of increasing signal-to-noise ratio.

### Time domain analysis: visual evoked potentials

In order to study the VEPs elicited by the different visual stimuli, we averaged the EEG signals of the epochs associated with each stimulus type and each stimulus contrast. Note that, for the calculation of the VEPs elicited by red-green and blue-yellow stimuli, both phases of the stimulation cycle were averaged together. The fast reversal rate of the achromatic stimulation induced steady-state VEPs, while the slower temporal frequency of the chromatic stimulation elicited transient VEPs. Steady-state VEPs are elicited when a repetitive visual stimulus is presented at a rate higher than 4 Hz, inducing a continuous sequence of oscillatory potential changes in the visual cortex [27]. Transient VEPs are elicited by abrupt visual changes with stimulation frequencies lower than 4 Hz and are typically comprised by a sequence of positive and negative deflections with return to pre-stimulus potential.

The signal strength of the achromatic responses was calculated based on the mean amplitude of the rectified wave within a stimulus cycle, that is, the area under the rectified VEP divided by the corresponding time interval (600 ms). The mean amplitude of the total response cycle was chosen as an alternative measurement to peak amplitudes given the difficulty in separating the individual peaks composing steady-state VEPs. Area under the curve (AUC) measurements of event-related potentials (ERPs) have been suggested as a valid alternative that diminishes the effect of trial-to-trial differences in ERPs’ latencies [28]. In here, AUC measurements were assumed to reflect the average of the amplitude of the bioelectrical signals elicited over occipito-parietal regions by the achromatic stimulation.

For the red-green and blue-yellow responses, we calculated the amplitudes and latencies of the peaks of the average signals by programming the Scan 4.5 software to automatically find the maximum of the waves within defined time windows: red-green, P1 maximum between 50 and 100 ms after stimulus phase reversal and P2 maximum between 100 and 185 ms; blue-yellow, P1 between 70 and 130 ms and P2 between 145 and 205 ms. The peaks with latencies between 250 and 350 ms present in the chromatic VEPs appeared as positive peaks in the grand averages of the NF1 group and as negative peaks in the grand averages of the control group. To quantify the group differences in this latter part of the signal without including assumptions about the polarity or number of peaks/components (as for the steady-state analysis), we calculated the mean area of the VEPs within the interval between 250 and 350 ms, that is, the area under the VEP waveform divided by the time interval of interest. We named this response late component (LC).

**Figure 2:**
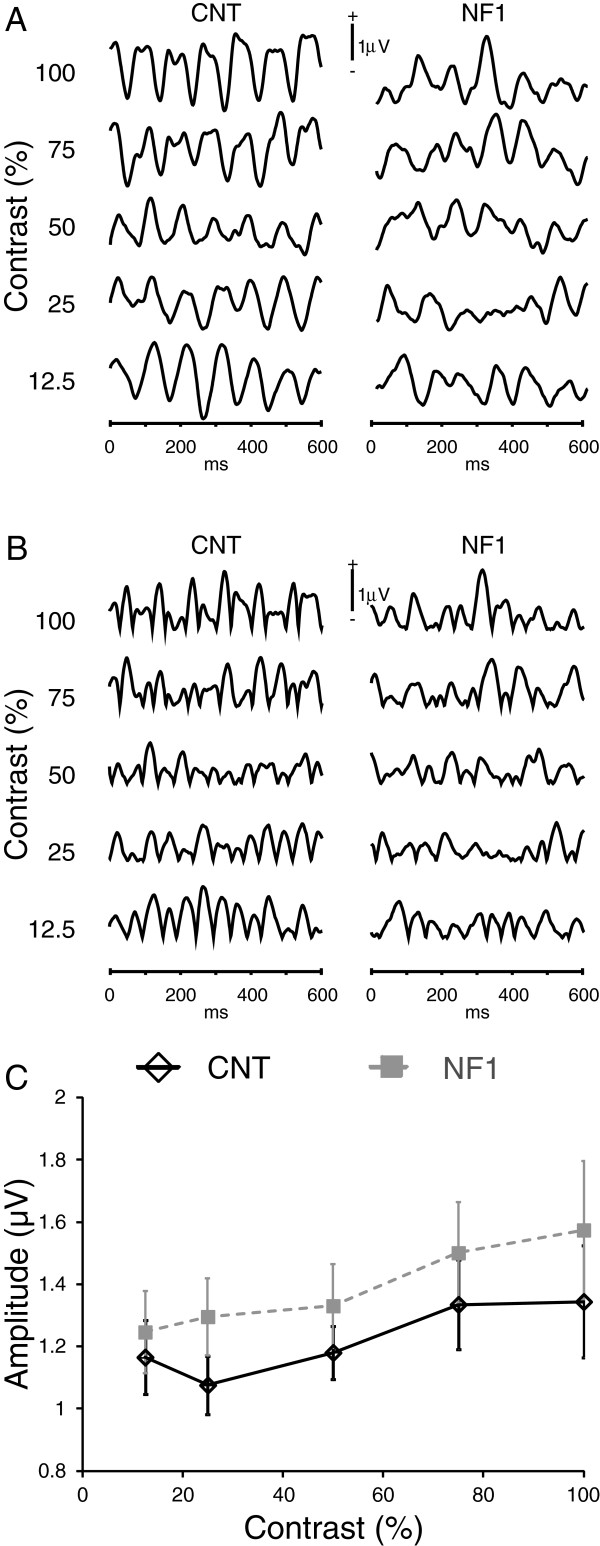
**Achromatic stimulation: neurofibromatosis type 1 (NF1) mean amplitude of the steady-state visual evoked potentials (VEPs) evoked by achromatic stimulation was not significantly different from control levels. (A and B)** Grand averages of (A) elicited VEPs and (B) of the rectified VEPs used for analysis for each stimulus contrast used (labeled on the left) for the control and NF1 groups. Positive voltage is up. **(C)** Contrast response function of the mean amplitude of VEPs for the control (black open diamonds) and NF1 group (grey squares). All data are represented as mean ± 1 standard error of the mean.

**Figure 3:**
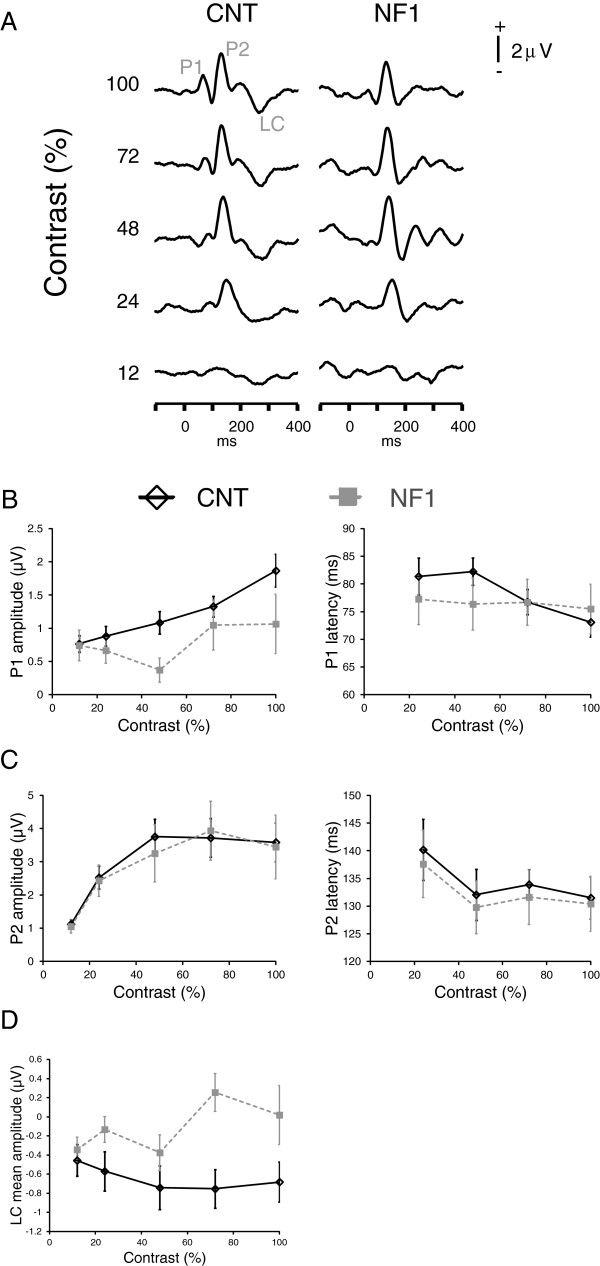
**Red-green stimulation: visual evoked potentials (VEPs) elicited by red-green stimulation, in patients with neurofibromatosis type 1 (NF1) and controls, revealed significant differences between the groups in the amplitude of late responses. (A)** Grand averages of the elicited VEPs for each stimulus contrast used (labeled on the left) for the control and NF1 groups. Positive voltage is up. **(B)** Contrast response functions for P1 amplitude (left) and latency (right). **(C)** Contrast response functions for P2 amplitude (left) and latency (right). **(D)** Contrast response function for the VEPs’ mean amplitude between 250 and 350 ms after phase reversal (LC). All data are represented as mean ± 1 standard error of the mean.

**Figure 4:**
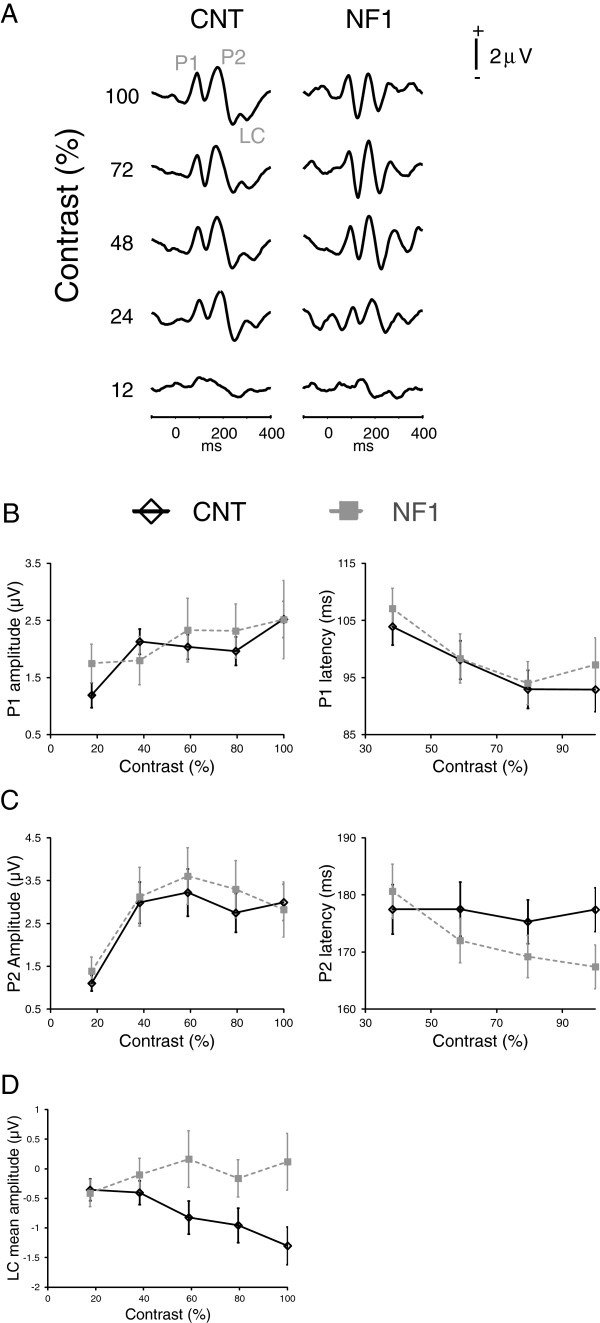
**Blue-yellow stimulation: visual evoked potentials (VEPs) elicited by blue-yellow stimulation, in patients with neurofibromatosis type 1 (NF1) and controls, revealed significant differences between the groups in the amplitude of the late responses between 250 and 350 ms after stimulus phase-reversal. (A)** Grand averages of the elicited VEPs for each stimulus contrast (labeled on the left) for the control and NF1 groups. Positive voltage is up. **(B)** Contrast response functions for P1 amplitude and latency. **(C)** Contrast response functions for P2 amplitude and latency. **(D)** Contrast response function for the VEPs’ mean amplitude between 250 and 350 ms after phase reversal (LC). All data are represented as mean ± 1 standard error of the mean.

For illustration purposes only, grand averages were created and low pass filtered with cutoff frequency of 30 Hz (Figures 2, 3, 4).

### Frequency domain analyses: amplitude of brain oscillations

During sensory processing, several types of EEG oscillatory signals can be differentiated by their degree of phase-locking to the stimulus [29]. Induced or non-phase-locked activity is correlated with stimulus processing but is not strictly phase-locked to its onset. Evoked or phase-locked activity is strictly phase-locked to the onset of the stimulus across trials, that is, it has the same phase in every stimulus repetition. Non-phase-locked activity is markedly reduced in the average of all trials (as its timing jitters from trial to trial). Thus, isolation of the phase-locked activity can be achieved by calculating the Fourier transform of the average ERP (that contains mostly phase-locked activity). Isolation of the non-phase-locked activity can be achieved by removing the VEP (average of all trials) from the raw EEG signal for each trial, according to the procedure used by Engell and McCarthy [30].

Hence, for the analysis of the amplitude of the non-phase-locked brain oscillations elicited by the achromatic stimulation, for each stimulus type and contrast, we removed the corresponding VEP from each 600 ms individual sweep. The amplitude spectrum was calculated by averaging across the fast Fourier transforms (10% Cosine window) of all the individual sweeps (minus the corresponding VEP). To calculate the amplitude of the phase-locked oscillations for each stimulus contrast, we calculated the fast Fourier transforms (10% Cosine window) of the VEP. This resulted in spectra with a frequency resolution of 1.7 Hz. For each amplitude spectra, we calculated the mean amplitude within the following frequency bands: delta (1.67 to 3.33 Hz), theta (5.01 to 6.66 Hz), alpha (8.32 to 11.65 Hz), beta (13.31 to 22.29 Hz), low gamma (25 to 43.25 Hz) and high gamma (55 to 80 Hz).

An equivalent procedure was used to determine the amplitude of phase-locked and non-phase-locked oscillations elicited by red-green and blue-yellow stimulation. For these signals, amplitude spectra were determined for the 500 ms sweeps used in the VEP analysis, resulting in spectra with a frequency resolution of 2 Hz. At each electrode, we calculated the mean spectral amplitude within the following frequency bands: delta (2 to 4 Hz), theta (4 to 6 Hz), alpha (8 to 12 Hz), beta (12 to 22 Hz), low gamma (24 to 44 Hz) and high gamma (54 to 80 Hz).

### Detection task: analysis of behavioral responses and calculation of the amplitude of pre-stimulus oscillations

The number of correct and incorrect responses and misses were expressed as a percentage of the total number of trials and calculated as follows. If the participants responded with a button press between 150 ms and 3,000 ms after the luminance of the fixation square changed, the trial was considered a detected trial (hit). If there was no response within this interval then the trial was considered a missed trial (miss). Of the detected trials, if the participants responded correctly to the type of polarity change in luminance (to a lighter square or a darker square) then it was considered a correct hit, if the participant responded with the wrong button then it was considered an incorrect hit.

For each type of stimulation (achromatic, red-green and blue-yellow) and for each type of response (correct hit and miss), we calculated the amplitude spectra by averaging the fast Fourier transforms (10% Cosine window) of each individual sweep containing the 1-second period just before luminance change. We did not analyze the incorrect trials as there were only a small number of these. In order to avoid significant differences in the number of sweeps of each condition (correct hits or misses), we enforced within-subject matching by removing sweeps from the condition with the highest number. Importantly, there were no group differences in the number of sweeps for each stimulation type (mean (standard deviation): achromatic stimulation, NF1 = 10 (5), CNT = 7 (4); red-green stimulation, NF1 = 25 (8), CNT = 20 (11); blue-yellow stimulation, NF1 = 21 (7), CNT = 18 (10)). For each amplitude spectrum (with 1 Hz resolution), we calculated the average amplitude in the alpha frequency band (8 to 13 Hz).

### Eyes-closed and eyes-open resting conditions

After eye blinking correction, filtering and manual rejection of artifacts, as described above, the EEG data from each 2-minute segment (eyes closed or eyes open) were divided into 4-second epochs. Epochs containing any remaining artifacts were automatically rejected on the basis of deflections with amplitude higher than 150 μV, as were the first 10 seconds in each condition. For each participant in each condition, amplitude spectra were calculated by averaging the fast Fourier transforms (10% Cosine window) of the single sweeps, with a frequency resolution of 0.25 Hz. The average spectral amplitude was calculated from the following six discrete frequency bands: delta (2 to 3.75 Hz), theta (4 to 7.75 Hz), alpha (8 to 12.75 Hz), beta (13 to 29.75 Hz), low gamma (30 to 45 Hz) and high gamma (55 to 80 Hz). Alpha peak frequency was determined as the frequency between 7 and 12 Hz at which the spectra reached a maximum amplitude value.

### Statistical analysis

All statistical analyses were performed with IBM SPSS Statistics, version 19, software (IBM Corporation, Armonk, NY, USA). We verified the normality assumption for the different parameters using the Shapiro–Wilk test. All measures were normally distributed except the number of incorrect trials obtained from the analysis of the visual detection behavioral data. For the normally distributed data, we used, as appropriate, ANOVA repeated measures analyses, parametric *t*-tests and Pearson’s correlation analyses. When the data did not meet assumptions of sphericity, the Greenhouse-Geisser correction was used. For the nonnormally distributed data, we used the Mann–Whitney test for comparisons between the groups.

All repeated measures ANOVAs included clinical group (NF1*,* control) as between-subjects factor. In addition, the following within-subjects factors were used: for the analysis of the VEPs’ amplitudes and latencies, stimulus contrast (five levels); for the analyses of the spectral amplitudes, spectral amplitude of the different frequency bands (six levels); for the analysis of behavioral data, visual stimulation type (achromatic, red-green, blue-yellow: three levels); for the analysis of pre-stimulus alpha amplitude, response type (hit*,* miss: two levels) and visual stimulation type (achromatic, red-green*,* blue-yellow: three levels); for analysis of the spectral amplitudes during the rest conditions, spectral amplitude of the different frequency bands (six levels) and the two conditions (eyes open, eyes closed: two levels).

## Results

### Time domain analysis: visual evoked potentials

First, we determined the contrast response functions of the mean amplitude of the steady-state VEPs elicited by achromatic stimulation with low-spatial, high-temporal frequency. All stimulus contrasts used elicited steady-state VEPs in both children and adolescents with NF1 and control participants (Figure 2A). However, group average VEPs appeared less stable in the NF1 group than in controls. Nevertheless, the amplitudes of VEPs did not show a significant effect of group or interaction between contrast and group (Figure 2B,C). As expected, there was a significant effect of stimulus contrast with mean amplitudes of the steady-state VEPs increasing with stimulus contrast (F_(2.2,73)_ = 5.0, *P* <0.01; Figure 2C).

Second, we characterized the VEPs elicited by chromatic stimulation. Relative isolation of the two chromatic channels was enhanced by using slow pattern reversal stimulation (2 rev/s reversal rate) that elicited transient VEPs.

The grand averages of the EEG responses elicited by red-green stimulation showed two positive peaks: one earlier peak (P1) at around 80 ms after stimulus phase reversal and one more prominent peak (P2) at around 120 ms (Figure 3A). The amplitudes of both peaks showed significant effects of stimulus contrast (P1: F_(2.9,97)_ = 5.3, *P* <0.01; P2: F_(1.6,54)_ = 19.8, *P* <0.001) but no significant interaction between contrast and group. P1 amplitude tended to be reduced in the NF1 group, however, this difference did not reach the significance level (F_(1,34)_ = 3.2, *P* = 0.08; Figure 3B). P2 amplitude levels were not significantly different between groups (Figure 3C). The latencies of both peaks did not show significant effects of stimulus contrast, interactions between contrast and group or effects of group (Figure 3B,C). Besides these two positive peaks, the group averages showed a broad negative peak apparent mainly in the control group at around 300 ms (LC) (Figure 3D). This negative potential was reduced in the NF1 group averages. The mean amplitude within a time window around 300 ms showed a significant effect of group (F_(1,34)_ = 6.4, *P* <0.05) with the control group presenting more negative values. There was also a marginally significant interaction between stimulus contrast and group (F_(2.5,84)_ = 2.4, *P* = 0.09), reflecting a larger difference between the groups for higher stimulus contrasts than for low stimulus contrasts (Figure 3D). The effect of stimulus contrast was not significant.

Similarly to the red-green response, the grand averages of the evoked responses elicited by the blue-yellow stimulation showed two positive peaks. However, these had longer latencies reflecting the slower response of the blue-yellow mechanism [31]: P1 (around 100 ms after phase reversal) and P2 (around 175 ms), visible both in the control and in the NF1 grand averages (Figure 4A). The amplitudes of P1 and P2 elicited by the blue-yellow stimulation showed significant effects of contrast (P1: F_(4,136)_ = 5.4, *P* <0.001; P2: F_(2.6,89.5)_ = 11.0, *P* <0.001; Figure 4B,C), no interactions between contrast and group and no effects of group (Figure 4B,C). P1 latency showed a significant effect of contrast (P1: F_(2.3,77.7)_ = 7.9, *P* <0.001), while the effect of stimulus contrast for P2 latency was not significant. The peak latencies did not show significant effects of group or significant interactions between contrast and group (Figure 4B,C). As for the VEPs elicited by red-green stimulation also the blue-yellow responses showed a broad negative potential more pronounced in the control group at around 300 ms (LC) (Figure 4A). The statistical analysis revealed a significant effect of group (F_(1,34)_ = 4.0, *P* = 0.05; Figure 4D), a significant interaction between stimulus contrast and group (F_(1.8,60)_ = 3.4, *P* <0.05) and no effect of contrast.

### Frequency domain analyses: amplitude of brain oscillations

**Figure 5:**
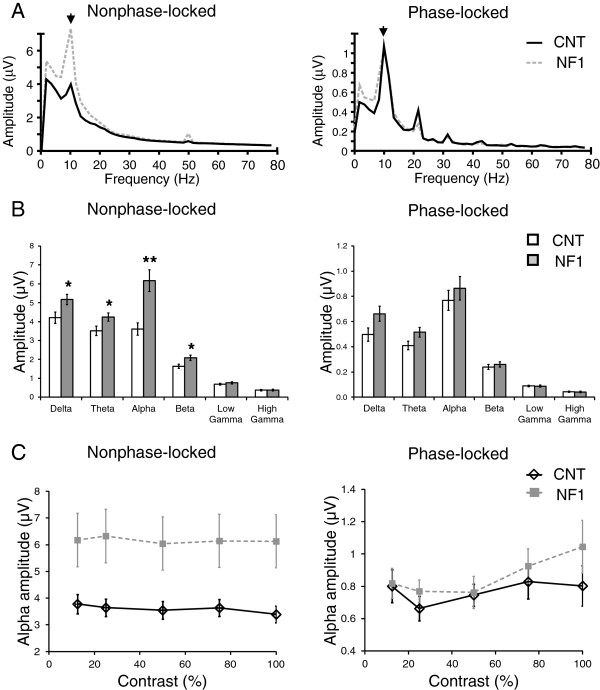
**Frequency domain analysis of the electroencephalographic (EEG) responses elicited by achromatic stimulation revealed significantly higher non-phase-locked alpha amplitude in children and adolescents with neurofibromatosis type 1 (NF1). (A)** Grand average amplitude spectra of neural oscillations non-phase-locked (left) and phase-locked (right) with the visual stimulation. **(B)** Control and NF1 average amplitude of each frequency band (theta, alpha, beta, low gamma and high gamma) for non-phase-locked (left) and phase-locked (right) oscillations. **(C)** Mean amplitude of non-phase-locked (left) and phase-locked (right) alpha as a function of stimulus contrast. All data are represented as mean ± 1 standard error of the mean. *P <0.05; **P <0.01.

Spectral analysis of the EEG responses elicited by achromatic stimulation revealed significant differences in visual processing between the two groups. The non-phase-locked oscillations presented a significant effect of group (F_(1,33)_ = 11.0, *P* <0.01) and a significant interaction between frequency band and group (F_(1.9,64)_ = 11.0, *P* <0.001). This significant interaction reflected the higher difference between the groups for the alpha band than the other frequency bands that can be observed in Figure 5A,B. On the other hand, phase-locked oscillations were not significantly different between the groups, reflected in a non-significant effect of group or interaction between group and frequency band.

As the alpha band showed the biggest difference between the groups, we studied the dependence of the alpha amplitude on stimulus contrast (Figure 5C). The amplitude of the non-phase-locked alpha oscillation decreased significantly with stimulus contrast (F_(2.6,85)_ = 2.9, *P* <0.05) and did not show a significant interaction between the effect of contrast and group, indicating that the difference in alpha amplitude between the groups was independent of stimulus contrast. The amplitude of the phase-locked alpha showed a significant effect of contrast (F_(2.9,94)_ = 3.4, *P* <0.05), very similar to the effect of contrast observed for the amplitude of the steady-state VEPs, as expected since the fundamental frequency of VEPs was 10 rev/s. We found no interaction between contrast and group and no effect of group.

**Figure 6:**
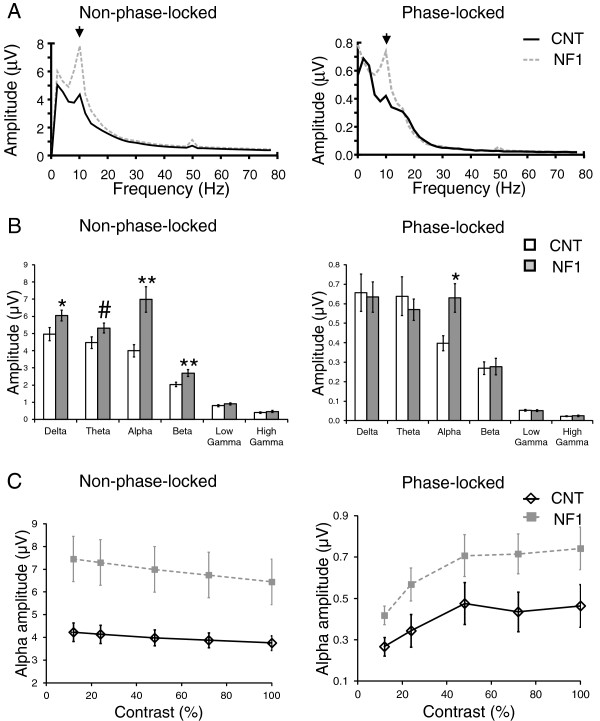
**Frequency domain analyses of the electroencephalographic (EEG) responses elicited by red-green stimulation revealed significantly higher amplitude of alpha oscillations in the responses of children and adolescents with neurofibromatosis type 1 (NF1) compared with control levels. (A)** Grand average amplitude spectra of the EEG responses non-phase-locked (left) and phase-locked (right) with the visual stimulation. **(B)** Control and NF1 average amplitude of each frequency band (delta, theta, alpha, beta, low gamma and high gamma) non-phase-locked (left) and phase-locked (right) with the visual stimulation. **(C)** Contrast response functions of control and NF1 mean amplitude of non-phase-locked (left) and phase-locked (right) alpha oscillations. All data are represented as mean ± 1 standard error of the mean. *P <0.05; **P <0.01; #*P* = 0.07.

**Figure 7:**
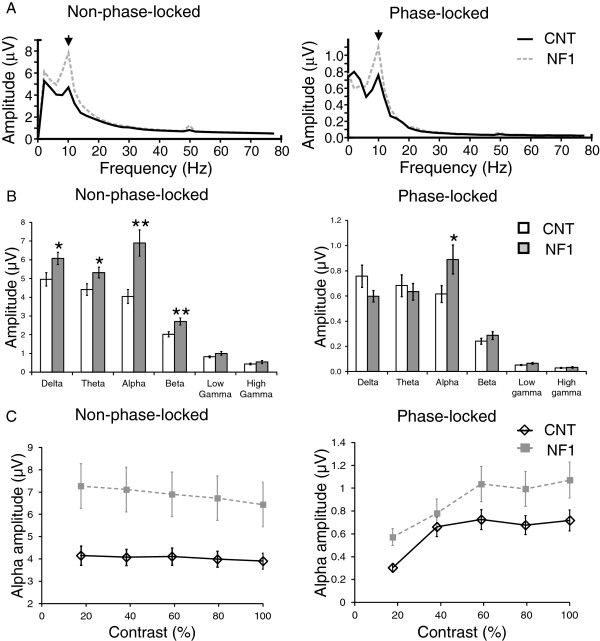
**Frequency domain analyses of the electroencephalographic (EEG) responses elicited by blue-yellow stimulation revealed significantly higher amplitude of alpha oscillations in the responses of children and adolescents with neurofibromatosis type 1 (NF1) compared with control levels. (A)** Grand average amplitude spectra of EEG responses non-phase-locked (left) and phase-locked (right) with the visual stimulation. **(B)** Control and NF1 average amplitude of each frequency band (delta, theta, alpha, beta, low gamma and high gamma) non-phase-locked (left) and phase-locked (right) with the visual stimulation. **(C)** Contrast response functions of control and NF1 mean amplitude of non-phase-locked (left) and phase-locked (right) alpha oscillations. All data are represented as mean ± 1 standard error of the mean. *P <0.05; **P <0.01.

Frequency domain analyses of the EEG responses elicited by chromatic visual stimulation also revealed significant differences between the alpha amplitudes of the two groups. The amplitude spectra of the responses elicited by red-green or blue-yellow stimulation are depicted in Figures 6A and 7A, respectively, showing higher alpha amplitude in NF1. The non-phase-locked oscillations showed a significant interaction between frequency band and group (red-green: F_(1.9,63)_ = 9.2, *P* <0.001; blue-yellow: F_(1.8,62)_ = 8.7, *P* <0.01) (emphasizing again the particular increase in the NF1 alpha band) and significant effects of group (red-green: F_(1,34)_ = 10.4, *P* <0.01; blue-yellow: F_(1,34)_ = 11.1, *P* <0.01). Post-hoc *t*-tests confirmed that the amplitude of the non-phase-locked alpha was significantly higher in the NF1 group when compared with control levels (red-green and blue-yellow: *P* <0.01; Figures 6B and 7B). Analysis of the amplitudes of the phase-locked oscillations revealed significant interactions between frequency band and group (red-green (marginally significant): F_(2.1,70)_ = 2.7, *P* = 0.07; blue-yellow: F_(2.5,86)_ = 4.1, *P* <0.05), once again consistent with the notion of increased NF1 oscillatory activity particularly in the alpha band (Figures 6B and 7B). The effect of group was not significant.

We also investigated the modulation of the alpha amplitude with stimulus contrast (Figures 6C and 7C). The amplitude of the non-phase-locked alpha decreased significantly with stimulus contrast (red-green: F_(1.9,65)_ = 34.3, *P* <0.001; blue-yellow: F_(2.6,90)_ = 18.4, *P* <0.001). There were also significant interactions between contrast and group (red-green: F_(1.9,65)_ = 4.5, *P* <0.05; blue-yellow: F_(2.6,90)_ = 5.7, *P* <0.01), reflecting a steeper decline of alpha amplitude with contrast in the NF1 group. In addition, for both types of chromatic stimulation, we found significant effects of group (red-green; F_(1,34)_ = 14.1, *P* <0.01; blue-yellow: F_(1,34)_ = 13.7, *P* <0.01) with higher non-phase-locked alpha amplitude in the NF1 group. For the phase-locked alpha, the amplitude increased significantly with stimulus contrast (red-green: F_(2.5,86)_ = 13.3, *P* <0.001; blue-yellow: F_(2.3,79)_ = 16.9, *P* <0.001) with significant effects of group (red-green: F_(1,34)_ = 8.3, *P* <0.01; blue-yellow: F_(1,34)_ = 4.5, *P* <0.05) and no interaction between the effects of contrast and group.

### Performance in the visual detection task and pre-stimulus alpha amplitude

**Figure 8:**
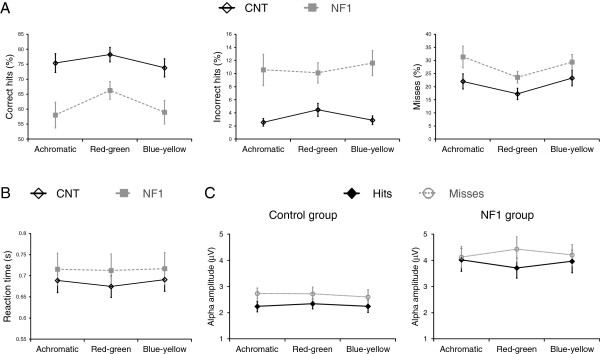
**Periods of high alpha are associated with lapses of attention both in controls and individuals with neurofibromatosis type 1 (NF1). (A)** Control and NF1 mean percentage of correct hits (left) and incorrect hits (middle) and misses (right) measured during achromatic, red-green and blue-yellow stimulation. **(B)** Control and NF1 mean reaction times of the correct hits, during achromatic, red-green and blue-yellow stimulation. **(C)** Mean pre-stimulus alpha amplitude for correct hits and misses. Error bars represent ± 1 standard error of the mean.

During EEG recording of visual responses, the participants were engaged in a visual detection task on fixation. The aim of the task was to help maintain fixation and to keep the participant’s attention stable throughout the EEG recording session. The task involved detection of a change in the luminance of the central fixation square that occurred unpredictably at random time intervals. Participants had to detect this brief change in luminance and report through button presses the type of polarity change (to a lighter square or a darker square). During the three types of visual stimulation, both control and participants with NF1 detected the luminance change correctly the majority of times (correct hits). However, in around 20% of trials the participants failed to respond to this visual event (misses). Repeated measures ANOVA showed a significant effect of group regarding the number of events responded correctly (correct hits: reduced in the NF1 group; F_(1,33)_ = 11.3, *P* = 0.002; Figure 8A) and a marginally significant effect of group regarding the number of missed trials (misses: increased in the NF1 group; F_(1,33)_ = 4.0, *P* = 0.06; Figure 8A). The number of incorrect responses were also found to be significantly different between the groups (incorrect hits: increased in the NF1 group; Mann–Whitney test *P* <0.01 for all stimulation types; Figure 8A). In addition, for the analyses of the number of correct hits and misses, we observed a significant effect of stimulation type (correct hits: F_(2,66)_ = 8.1, *P* = 0.001; misses: F_(1.6,53.7)_ = 8.1, *P* = 0.002). This effect reflected the higher number of correct hits and lower number of misses during red-green stimulation than during the two other types of stimulation. There was no interaction with group suggesting a similar effect of stimulation type in both groups. Correct hits reaction time was not significantly different between the groups (Figure 8B).

The higher number of missed and incorrect responses observed in children and adolescents with NF1 indicates difficulties in sustained attention, a cognitive function known to be affected in NF1 [32]. These errors might be associated with momentary lapses of attention characterized by high alpha amplitude [18]. Thus, we were interested in determining if, in our cohorts, the missed trials were related to higher pre-stimulus alpha amplitude. Repeated measures analysis revealed that the alpha amplitude during the 1-second period immediately before the visual cue was significantly higher for missed trials than for detected trials (significant effect of response type F_(1,33)_ = 7.7, *P* <0.001; Figure 8C). There was no significant interaction between the effects of response type and group suggesting that a similar modulation of alpha was associated with misses and hits in both groups. We found no significant effect of visual stimulation type and a marginally significant three-way interaction, stimulation type x response type x group (F_(2,66)_ = 2.6, *P* = 0.08), reflecting the fact that in the NF1 group the effect of alpha amplitude on response type was bigger during red-green trials than during blue-yellow or achromatic trials and that this difference was not observed in the control group (Figure 8C). As expected, there was a significant effect of group (F_(1,33)_ = 13.6, *P* = 0.001) with higher alpha amplitude in the NF1 group.

These findings suggest that alpha oscillations predict behavioral performance in both controls and individuals with NF1.

### Alpha oscillations during idle periods with eyes closed and with eyes open

**Figure 9:**
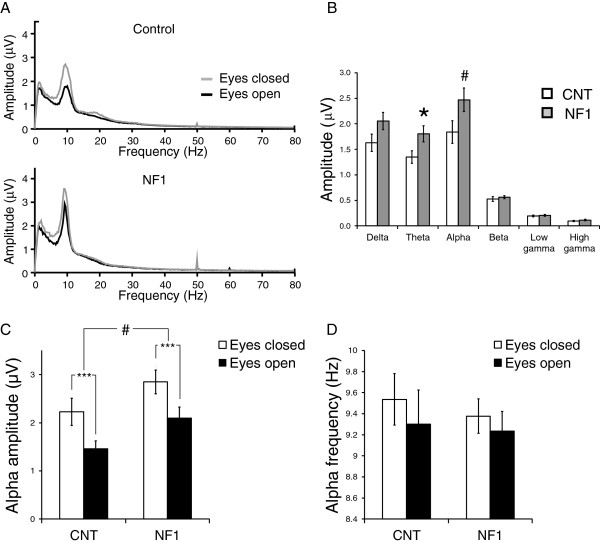
**Alpha amplitude during eyes open or eyes closed resting conditions was higher in children and adolescents with neurofibromatosis type 1 (NF1) than controls. (A)** Control (top) and NF1 (bottom) amplitude spectra of the electroencephalographic (EEG) recordings during eyes closed (grey lines) and eyes open (black lines) resting conditions. **(B)** Control and NF1 mean amplitude of each frequency band: average across the eyes closed and eyes open conditions. **(C)** Control and NF1 mean amplitude within the 8 to 12.75 Hz alpha band, during eyes closed and eyes open. **(D)** Control and NF1 mean alpha peak frequency during eyes closed and eyes open. All data represented as mean ± 1 standard error of the mean. *P <0.05; ***P <0.001; #*P* = 0.07.

In order to determine if oscillatory activity at rest was altered in NF1, we recorded the EEG signal during periods of rest with eyes closed alternating with periods of fixation with eyes open in a subset of children and adolescents with NF1 and controls. In normotypicals, the amplitude of alpha brain oscillations is higher during awake eyes-closed idle states and reduces upon the opening of the eyes [33]. Repeated measures analysis showed a significant effect of group (F_(1,24)_ = 5.3, *P* <0.05) and a significant interaction between the amplitude of the frequency bands and group (F_(1,1.9)_ = 3.3, *P* = 0.05) indicating that the differences between the groups were more pronounced for some bands than for others. Post-hoc *t*-tests revealed significant differences between the groups for the theta band (*P* = 0.03) and marginally significant for the alpha band (*P* = 0.07) with NF1 amplitudes higher than control levels (Figure 9A,B). As expected, the analysis revealed a significant effect of condition (F_(1,24)_ = 61.6, *P* <0.001) but no interaction between condition and group indicating that the amplitudes of the oscillations were modulated by eyes opening in a similar way in both groups (Figure 9C shows the modulation of alpha amplitude). Thus, during idle states, the amplitudes of alpha brain oscillations were higher in NF1.

We also studied the alpha peak frequency as it is related to mental state and cognitive abilities [34]. However, we found no significant differences between the groups (Figure 9D), no effect of condition and no interaction between condition and group.

### Effect of IQ and ADHD co-morbidity

The mean IQ scores of children and adolescents with NF1 are commonly found to be slightly lower than average [1]. It is of interest to try to determine if the differences in cortical function observed in this study relate directly to deficits in general cognitive function (IQ). We therefore performed, in the NF1 group, correlation analysis between IQ and the neurophysiological measures that were significantly different between the groups, that is, amplitude of late VEPs elicited by chromatic stimuli, amplitude of non-phase-locked alpha oscillations for each type of visual stimulation and theta and alpha amplitude during the eyes closed and eyes open conditions. However, none of these measures of brain activity correlated significantly with IQ. Notably, this finding also indicates that group IQ differences are not a major factor in our results. Indeed, when we compared two subsamples of patients with NF1 and control participants with matched IQ (n = 6 for each group), we still observed significant differences between the groups in the average non-phase-locked alpha amplitude for the three types of visual stimulation (*t*-tests, *P* <0.01).

Patients with NF1 present an enhanced predisposition for ADHD. When the ADHD diagnostic criteria are met, these patients tend to show a more severe cognitive phenotype than patients with NF1 without ADHD [35]. Our findings might have been exacerbated by the inclusion of patients with NF1 and ADHD comorbidity (n = 4). However, after excluding the data from patients with NF1 and ADHD, we still observed similar patterns of EEG abnormalities. Chromatic ERPs showed significant effects of group in the late components between 250 and 350 ms after stimulus reversal (red-green: F_(1,30)_ = 7.1, *P* <0.05; blue-yellow: F_(1,30)_ = 4.2, *P* <0.05). The significant enhancement of alpha amplitude was still observed in the non-phase-locked oscillations (achromatic: F_(1,29)_ = 22.7, *P* <0.001; red-green: F_(1,30)_ = 16.4, *P* <0.001; blue-yellow: F_(1,30)_ = 16.9, *P* <0.001) and, during chromatic stimulation, in the phase-locked oscillations (red-green: F_(1,30)_ = 10.9, *P* <0.01; blue-yellow: F_(1,30)_ = 5.3, *P* <0.05). During rest eyes open/eyes closed conditions, patients with NF1 without ADHD (n = 9) presented significantly higher amplitudes of theta and alpha oscillations (*t*-tests, *P*s <0.05).

## Discussion

Our results showed a specific enhancement of alpha oscillations both during visual stimulation and during rest periods with eyes closed and eyes open in children and adolescents with NF1. Alpha reactivity was normal in the NF1 group showing a decrease with eyes opening when compared with eyes closed condition. Furthermore, the influence of spontaneous modulation of alpha amplitude in visual performance, observed in the analysis of the visual detection task, was similar between the groups, with higher alpha related to a higher probability of missing the target in both groups. These observations suggest that the alpha rhythm in the NF1 group, although abnormally enhanced, is functional. In addition, this study showed that early visually evoked responses to low-level visual stimuli were not significantly affected, whereas anomalies were apparent in long-latency evoked responses to chromatic stimuli, suggesting deficits in later stages of neural processing.

Most of the individuals with NF1 included within this EEG study were also included in a previous study of contrast sensitivity where we found evidence for low-level visual deficits [8]. Thus, although a direct link remains to be shown, the abnormal cortical responses described here may be associated to visual problems.

Source localization studies have shown that early VEPs (around 100 ms after stimulus onset) arise from a high number of cortical sources, including V1, V2, V3 and lateral occipital areas [36-38]. Our present study suggests that these early visual responses were not significantly affected (although the early red-green peak at around 80 ms showed a marginally significant reduction in amplitude in the NF1 group). Only long-latency evoked responses showed significant group differences. As the limited scalp cover used in our study hindered source localization, it is not possible to determine which brain areas underlie the anomalies observed. Differences could include abnormal re-activation of early occipito-parietal visual areas [37] or abnormal higher level visual processing across the occipito-temporal or occipito-parietal cortex.

In addition to atypical VEPs, abnormal brain oscillations could also be related to deficits in visual processing. Previous studies have shown that neural responses to periodic visual stimuli resonate with endogenous brain rhythms at around 10 Hz (the frequency of the alpha oscillations), that is, visual stimulation with temporal frequency around 10 Hz, or its harmonics/sub-harmonics, elicit cortical responses that show phase-locked spectra with a marked peak around 10 Hz [39-42]. This suggests that visual cortical networks have a natural tendency to oscillate at the alpha frequency. Accordingly, our phase-locked spectra show peaks in the alpha frequency in response not only to the 10 rev/s achromatic stimulation but also in response to the 2 rev/s chromatic stimulation. The phase-locked alpha evoked by the 10 rev/s stimulation was not significantly different across the groups suggesting that the capacity of the NF1 neural circuits to oscillate at this stimulation frequency is not affected. In contrast, the NF1 phase-locked 10 Hz response was abnormally high in response to 2 rev/s visual stimulation. This finding suggests that the NF1 cortical neurophysiology is atypical, presenting an abnormally high propensity to oscillate at the alpha frequency even when the stimulation frequency does not fully drive the rhythm. This fact might have important perceptual consequences. Indeed, alpha phase-locking by rhythmic sensory stimulation has been shown to affect visual perception [42,43]. It will be important to determine if enhanced alpha phase-locking affects visual perception in individuals with NF1.

Abnormal non-phase-locked alpha oscillations could also be related to deficits in visual processing. Periods of high alpha amplitude have been associated with poor detection of threshold stimuli [44-46], and a causal link has been established between high alpha amplitude and deficient visual processing [47]. In addition, sensory processing is also associated with modulation of non-phase-locked oscillatory activity. In the visual cortex, visual stimulation induces an increase in non-phase-locked gamma amplitude and a decrease in non-phase-locked alpha amplitude [48]. Accordingly, we observed in both groups of participants, a decrease in non-phase-locked alpha amplitude with increasing stimulus contrast, that is, with increasing stimulus saliency. This mechanism of stimulus induced alpha suppression was thus not affected in the NF1 group. High non-phase-locked alpha amplitude might instead reflect difficulties in self-regulation of attention and arousal levels [49,50]. This hypothesis is compatible with our previous fMRI findings where we observed impaired deactivation of default mode network areas during rhythmic visual stimulation in individuals with NF1 [7]. Activity in the default mode network correlates positively with the amplitude of the alpha rhythm and also with attention lapses [51,52].

An intriguing finding of this study was that NF1 achromatic VEPs were not significantly different from the control VEPs, although average responses appeared more variable and with lower amplitude. This result might be a consequence of reduced VEP synchronization across subjects in these patients. Interestingly, this hypothesis would be compatible with enhanced amplitude of non-phase-locked alpha in the NF1 group.

Average IQ of the participants with NF1 that were included in this study was lower than control levels. This difference is in agreement with the downward shift of IQ observed in individuals with NF1 [1]. Basic sensory processing is not thought to be related to IQ and therefore, it is unlikely that IQ levels could have influenced our findings. Indeed, our result showing that two subgroups of patients and controls matched for IQ still differed significantly in alpha amplitude measured during visual stimulation further supports the idea that alpha dysfunction is a characteristic of patients with NF1 regardless of IQ. This is also consistent with our correlation analysis.

Children and adolescents with NF1 show increased probability of presenting ADHD symptoms [1]. As alpha oscillations are related with visual attention and task engagement, the differences observed might have been accentuated by the inclusion of patients with ADHD co-morbidity. However, when we excluded these patients from the analysis we still observed significant differences between the groups in alpha amplitude, both during visual stimulation and rest, suggesting that enhanced alpha is related with NF1 and not to the ADHD comorbidity present in some patients. Unfortunately, the small number of patients with NF1 and ADHD included did not allow for a comparison between patients with and without ADHD. This comparison would be important to determine if ADHD in patients with NF1 leads to a different EEG profile from the one observed here.

The cause of the abnormal alpha rhythm observed in children and adolescents with NF1 is unclear. Interestingly, the thalamus plays a strategic role in the generation of normal alpha rhythms [53] and presents abnormal structure and metabolism in NF1 [2,3,5], raising the hypothesis that thalamic dysfunction could underlie the alpha phenotype.

## Conclusions

Here, we have described two anomalies in cortical function in children and adolescents with NF1 that may be related with the visual deficits previously described in this disorder: 1) a specific enhancement of alpha brain oscillations that may be related to difficulties in attention allocation; and 2) abnormal long-latency VEPs indicating deficits in high-level processing of visual stimuli. These findings suggest that visual deficits in these patients are not likely to emerge due to problems in low-level stimulus processing but rather might be related to deficits in higher order functions such as allocation of attention.

## Abbreviations

CIE: Commission Internationale de l’Éclairage; cpd: Cycles per degree; CRT: Cathode ray tube; EEG: Electroencephalogram; ERP: Event-related potential; FLAIR: Fluid-attenuated inversion recovery; fMRI: Functional magnetic resonance imaging; IQ: Intelligence quotient; L: Long-wavelength; LC: Late component; M: Middle-wavelength; MPRAGE: Magnetization-prepared rapid acquisition of gradient echo; NF1: Neurofibromatosis type 1 rev/s: Reversals per second; S: Short-wavelength; VEP: Visual evoked potential; WISC: Wechsler Intelligence Scale for Children; ADHD: attention deficit hyperactivity disorder.

## Competing interests

The authors declare that they have no competing interests.

## Authors’ contributions

MJR, OCA, EDS and MCB conceptualized and designed the study. MJR and OCA carried out the EEG acquisition sessions. MJR analyzed the data and drafted the manuscript. MJR, OCA, EDS and MCB contributed to the interpretation of the data and manuscript writing. FR and JS contributed with patient recruitment and patient characterization. All authors read and approved the final manuscript.
